# Supplementary material for: Are carbon emissions trading and green financial instruments synergistic? -Comprehensive quantitative research based on content analysis
Source: PLoS One. 2024 Mar 7;19(3):e0298601. doi: 10.1371/journal.pone.0298601 (PMC10919601; doi:10.1371/journal.pone.0298601)
Supplement: S1 Data — (ZIP) [file pone.0298601.s001.zip › Data/地级市Z_F工作报告.zip!/╜¡╦╒/┴1⁄4╘╞╕█╩╨/└┤╫╘╣1⁄2╓┌║┼í╛┬φ┐╦╩2╛▌═°í┐_2014.docx]

各位代表：

　　现在，我代表市人民政府向大会作工作报告，请予审议，并请各位政协委员提出意见。

　　2013年工作回顾

　　过去的一年，在市委的正确领导下，我们深入贯彻落实科学发展观，团结和依靠全市人民，抢抓国家战略叠加的重大机遇，紧紧围绕　五个加快、十大突破和市十三届人大二次会议确定的各项目标任务，统筹做好稳增长、调结构、促改革、惠民生各项工作，在推动科学发展、跨越发展的道路上迈出了坚实的步伐。

　　一、稳增长、促转型，在应对复杂局面中全市经济保持稳中有进态势。在经济下行压力加大的情况下，我们采取有力政策措施，推动全市经济实现平稳较快增长。尤为可喜的是，结构调整步伐加快、亮点增多。

　　多措并举稳增长。强化运行调度，加强对经济形势的研判应对，狠抓重大事项重点工程督查推进，完善挂钩帮办、集中会办机制，及时协调解决重点难点问题。扩大有效投入，突出抓好500个市级重大产业、基础设施、民生建设项目，虹港石化、亚新钢铁等112个亿元以上工业项目竣工投产，全市完成全社会固定资产投资1664亿元。加强工业技改，财政投入亿元专项资金，鼓励引导企业推广应用新技术、新工艺、新装备和新材料，完成技改总投资480亿元，占规模以上工业投资的57%。重抓招商引资，精心组织赴日韩、台湾、北京、苏南及陆桥沿线城市开展大型招商推介活动，实际利用外资9.4亿美元，内联客方到位资金767亿元。预计全市地区生产总值增长12.3%，公共财政预算收入增长14.7%，规模以上工业增加值增长14.5%，全社会固定资产投资增长23%，社会消费品零售总额增长13.5%，居民消费价格涨幅低于省控标准。主要经济指标保持在合理区间之内，多数指标增幅位居全省前列。

　　加快转型调结构。推进工业集聚发展，装备制造业产值率先突破千亿，高新技术产业实现产值1500亿元。炼化一体化项目获得国家发改委批准并已着手开展前期工作，连云港跻身国家规划建设的七大世界级石化产业基地。六大重点园区实现销售收入3200亿元，海州区被认定为国家火炬计划特色产业基地。全市销售收入过50亿元企业达到17家，较上年增加7家，新海石化过200亿元。中小企业园和工业集中区开工建设标准厂房264万平方米，签约企业359家。加快发展现代服务业，27个集聚区全面推进，50个重点项目完成投资133.6亿元，东海水晶文化创意产业园入选首批江苏文化科技产业园，金港湾物流园区获批省级现代服务业集聚区。花果山成功创建全国旅游标准化试点景区，云台山国家森林公园开园。港口完成货物吞吐量2亿吨、集装箱548.8万标箱，铁水联运完成25万标箱。民航完成旅客运输量56.4万人次。招商银行连云港分行、江南农商行赣榆支行开业，全市各类融资额比年初新增310亿元，其中直接融资115亿元。大力实施农业现代化工程。新增设施农业15万亩、设施渔业6.7万亩，建成万亩菜篮子基地3个，高公岛获批国家一级渔港。新增市级农业园区10家、省级农业龙头企业13家。新增绿色农产品品牌　48个。农产品出口4.1亿美元。开展农田水利设施建设年活动，完成水利投资20亿元。新增高标准农田20万亩。农业机械化水平达到76.5%。

　　搭建载体促创新。荣获全国科技进步先进市，获批国家知识产权试点城市。全市新增国家级高新技术企业30家，累计达104家。新增国家科技重大专项2项，累计达56项。高性能碳纤维、热毒宁注射液获评国家战略性创新产品，中蓝连海、豪森分获国家科技进步一、二等奖，恒瑞、康缘荣获中国专利金奖。质量强市战略深入推进，新增中复连众等中国驰名商标3件、省级名牌产品24个。重大创新平台建设加快，建成国家级企业重点实验室、工程技术中心8家，市工业展览中心建成使用，新医药产业园公共服务平台主体完工。中科院能动中心部分装置投入实验，省海洋资源研究院建成功能材料重点实验室。市开发区、东海县获批省级科技金融示范区。新增国家级博士后科研工作站3家，累计引进国家千人计划人才14人。

　　二、打基础、求突破，在落实国家战略中港城跨越发展迈上新的起点。一年来，我们积极对接落实政策，加快完善基础设施，大力推进载体建设，致力将国家战略聚焦优势转化为现实发展优势。

一体两翼组合大港基本形成。主体港区30万吨级航道一期工程全面建成，码头泊位和集疏运体系不断完善，煤炭转运系统建设、主次干道改造等整治出新工程完工，新国际客运站主体建成，港口功能形象明显提升。徐圩港区10万吨级航道建成通航，实现开港运营。赣榆港区集装箱航线正式开通，灌河港区燕尾作业区开港运营。保税物流中心二期建成投用。属地申报、属地验放跨区域通关新模式启动试行，口岸通关便利化水平进一步提升，连云港口岸申报货物进出口总值达250亿美元。

　　国际性海滨城市建设全面加速。市区建成区面积扩大至150平方公里，全市城市化率提高到57%。制定实施城市规划、建设、管理3个导则。新海新区科技创业城一期、新世界文化城等项目建设快速推进，城市副中心日益显现。连云新城23条主次干道和27座桥涵全面建成，一批功能性基础设施加快建设，新城框架初步呈现。南部新城开发建设快速启动，各项规划不断完善，重点工程推进有力。4个区级城市组团建设有序推进。全市完成旧城改造500万平方米，一批背街小巷整治出新，东方花园等10个低洼片区完成改造。海滨大道市区段基本实现贯通。成功修建连云港老街、新浦民主路等历史文化特色街区。花果山大道、朝阳路等13条道路改造升级。沭新渠送清水工程实现通水，蔷薇湖生态湖区开工，第三水厂扩建工程建成投产。农村环境不断改善，浦南镇、新坝镇等第一批30个试点镇村建设加快，第二批65个试点镇村规划编制完成，建成农民新社区23个。

　　重大基础设施建设加快推进。临海高等级公路基本建成通车，310、242、324等国省干线公路全面推进，盐河航道整治完工。连盐铁路开工建设，连淮扬镇铁路获批立项。新机场选址通过初审。连云新城海堤达标、徐圩新区防洪除涝等重点水利工程加快建设。500千伏伊芦变扩建等12个电网工程建成投用。信息通讯开始步入4G时代。

　　东中西区域合作示范区先导区初步形成五纵五横路网框架和一区七园发展格局。

　　对外合作开放开创新局面。在中哈两国元首共同见证下，连云港与哈国铁路公司签署共建过境运输出海通道和中转分拨基地协议，上合组织成员国总理第十二次会议将连云港确定为共用出海口，丝绸之路经济带东方桥头堡地位得到进一步确立。成功举办连云港之夏旅游节、丝绸之路经济带合作发展论坛、第12届泛黄海中日韩经济技术交流会议等重大文化经贸活动，进一步提升了我市的知名度和影响力。

　　三、办实事、惠民生，在加快社会事业发展中群众得到越来越多实惠。坚持发展为了人民，财政预算民生支出力度加大，保障和改善民生六大体系加快构建，十大民生工程顺利实施，赣榆、东海通过省全面小康达标验收，人民群众沐浴到更多基本公共服务的温暖，享受到更多改革发展的成果。

　　围绕学有所教，坚持教育优质均衡发展，教育现代化建设取得显著成效。我市成为苏北唯一的教育现代化市级示范区。全面完成国家、省校安工程三年规划任务，改造中小学校舍51.4万平方米，市区建成投用中小学校6所，全市90%以上高中段学生在三星级以上普通高中和职业学校就读。淮海工学院3个硕士点通过国家验收，康达学院建成招生。扎实做好扶贫助学工作，外来务工人员子女就学享受同城待遇。

　　围绕劳有所得，拓宽渠道、强化保障，城乡居民收入持续增长。完成各类职业技能培训33万人，新增城镇就业8.1万人、下岗失业人员再就业2.1万人、农村劳动力转移就业3.7万人。社会保险实现市级统筹，职工五项保险参保人数净增16.7万人，被征地农民社会保障实现全覆盖。实施新一轮扶贫开发，10万低收入人口、100个经济薄弱村实现脱贫。城镇居民人均可支配收入达到2.66万元，增长9.2%；农民人均纯收入首次突破万元，增长10.6%。

　　围绕病有所医，着力提升基本公共卫生服务能力，群众看病难、看病贵问题得到缓解。公办基层医疗卫生机构全部配备使用基本药物，并实现零差率销售。在全省率先试行城镇居民重大疾病按病种收付费，完成新农合支付方式改革。市一院新海新区分院开工建设，市直属医院全部建成三级医院，创建省级卫生镇村45个。免费婚检率达73.8%，免费孕前优生健康检查实现全覆盖。

　　围绕老有所养，加快配套设施建设，养老服务和社会救助水平稳步提高。建成居家养老服务中心269个，公办示范性养老机构实现全覆盖。企业退休人员养老金九连调，城乡低保户、失独家庭、残疾人等特殊群体供养标准稳步提高。市社会福利中心建成投用，市民政公共服务中心等便民利民设施加快建设。

　　围绕住有所居，增加投入、创新机制，住房保障覆盖范围不断扩大。统筹推进各类保障性住房建设，开展经济适用房共有产权制度创新试点，新开工保障性住房8374套，发放廉租住房补贴2018户、住房公积金贷款25亿元，2917户棚户区居民乔迁新居。

　　围绕娱有所乐，大力推进文化惠民，群众文体活动日趋多样化。报业、广电、演艺三个文化集团成功挂牌。举办在海一方系列文化演出活动500多场。社区文化中心标准化建设成为国家第一批公共文化服务示范项目。新闻出版市场繁荣有序。全民健身馆竣工，市体育中心一场三馆全面投入使用。妇女儿童活动阵地在全省率先实现县、镇、村三级全覆盖。

　　同时，我们切实加强社会管理和生态建设，经济社会呈现出协调发展良好局面。认真做好法治连云港、平安连云港创建，推进依法行政、全民守法，深入开展三解三促、大接访和百案化解攻坚活动。安全生产检查常态化、制度化，连续九年被评为省安全生产目标考核一等奖。食品药品安全工作得到加强。新增造林26万亩，市区新增绿地437公顷，绿化覆盖率达到41%。大力开展县乡河道疏浚和村庄河塘整治。25万农村居民饮水安全问题得到解决。乡镇垃圾中转站实现全覆盖。秸秆综合利用与禁烧成效明显。完成省下达节能降耗任务。外事侨务、港澳台事务得到加强，民族宗教、防震减灾、科普、气象、档案、史志、红十字、关心下一代和老区开发事业取得新进步，双拥共建、国防动员、民兵预备役和优抚安置工作取得新成绩。

　　一年来，我们认真落实中央和省、市委改进工作作风、密切联系群众各项规定，以反对四风、服务群众为重点，深入开展五治作风整顿、效能港城政风行风评议活动，切实加强政府自身建设。全年共受理涉及软环境和行政效能问题投诉1182件，办结率97%；政府服务热线一级平台共受理案件26.1万件，处置率达99%；清理各类规章制度1263条。自觉接受人大及其常委会的法律监督、工作监督和人民政协的民主监督，办结人大代表建议130件、政协委员提案319件。严格执行国务院约法三章规定，强化审计监督和行政监察，压缩一般性行政支出5%，公车购置数量下降40%，廉政勤政建设富有成效，服务改革发展的水平显著提升。

　　各位代表，过去的一年，我市大事多、喜事多。能够取得这样的成绩，得益于市委科学决策、正确领导，得益于全市人民同心同德、艰苦奋斗，得益于人大、政协有效监督、大力支持，得益于民主党派、工商联、人民团体和无党派人士肝胆相照、携手合作，得益于广大客商和企业家共克时艰、共创大业，得益于驻连部队、武警官兵、公安干警牢记使命、忠诚履职，也得益于老干部、老同志的关心、理解和支持。在此，我代表市人民政府向全市人民，向所有关心连云港发展、参与连云港建设的各界人士，致以崇高的敬意，表示衷心的感谢！

　　在肯定成绩的同时，我们也清醒地认识到，连云港正处于加快发展、转型发展的关键时期，经济社会发展中还存在不少困难和问题。主要是：经济企稳回升基础不牢，部分行业和企业生产经营困难；资金、人才等要素制约愈加凸显，资源环境压力日益加重；公共服务体系不尽完善，民生工作与群众期盼还有一定差距；机关作风仍需进一步改进，发展环境有待进一步提升。我们将以对连云港发展高度负责的态度，加快解决这些问题，更加努力做好工作，决不辜负全市人民期望！

　　今年是全面深入贯彻落实党的十八届三中全会精神的开局之年，是实施十二五规划和沿海开发战略的关键之年，是我市以县为单位全面建成小康社会之年，也是我市被确定为全国首批沿海开放城市30周年。做好今年的工作，意义重大而深远。

　　　当前，我们发展面临的环境和条件都在发生深刻的变化，风险与机遇同在，挑战与发展并存。尽管国际政治经济形势仍较复杂严峻，国内经济面临着一定的下行压力，但我国经济长期向好的基本面没有改变，特别是十八届三中全会对全面深化改革作出总体部署，将进一步激发发展的内生动力和活力。同时，随着丝绸之路经济带建设加快推进，四大国家战略深入实施，连云港发展的政策叠加效应将持续释放。近年来港产城融合发展打下的坚实基础，也为我们在更高平台上推进发展提供了有力支撑。我们将坚定信心，把握机遇，真抓实干，奋力把港城科学发展、跨越发展不断推向前进。

　　今年政府工作的指导思想是：全面贯彻落实党的十八大和十八届二中、三中全会精神，按照中央、全省经济工作会议和省、市委全会的部署，抢抓一带一路建设重大机遇，坚持稳中求进工作总基调，以改革创新促发展，以扩大开放求突破，着力加快转型升级，着力推进城乡统筹，着力抓好民生改善，着力建设生态文明，确保实现以县为单位全面建成小康社会，推动国际性海港中心城市建设迈进新阶段。

　　主要预期目标是：地区生产总值增长11%，公共财政预算收入增长12%，全社会固定资产投资增长22%，社会消费品零售总额增长13%，注册外资实际到账10亿美元，引进内联客方到位资金850亿元，城镇居民人均可支配收入增长10%，农民人均纯收入增长11%，居民消费价格保持相对稳定，节能减排完成省定目标。

　　围绕以上目标要求，我们将重点做好以下工作：

　　一、弘扬创新精神，积极突破重点领域改革。按照中央和省、市委的部署要求，立足连云港发展的阶段性特征，以更大的勇气和魄力推进改革，为全市又好又快发展注入强大动力和活力。

　　坚持市场化方向，深化经济体制改革。更加尊重市场规律，发挥市场在资源配置方面的决定性作用，为各类市场主体营造公平竞争环境。出台扶持民营经济发展意见，放宽准入，降低门槛，集成政策，推动全民创业，大力促进民营经济发展。突出科技、金融、旅游等方面的改革创新，建立完善统筹推进服务业发展的体制机制。按照财权事权统一原则，完善市与区两级财政管理体制，推进全口径预算编制，促进预算编制、执行、监督、绩效四位一体。以深化国资改革带动国企改革，优化国有资产管理体制，规范投融资平台运作，强化风险管理与控制。稳步推动资源环境价格改革，实施居民生活阶梯式气价，探索利用市场化手段推进节能减排、资源有偿使用和生态环境补偿机制。突出抓好行政审批制度改革，打好全面深化改革第一仗，最大限度激发市场活力。

　　坚持一体化导向，深化城乡统筹改革。大力培育新型农业经营主体，推进农村经营方式创新。加快农村土地确权登记颁证工作，开展集体产权股份合作制改革，发展农村产权交易市场，探索建立土地承包经营权流转价格市场决定机制。扶持发展村镇银行、农村小额贷款公司，规范发展农村资金互助合作，扩大三农贷款有效抵押物范围。探索财政贴息，引导金融机构在镇村开展信贷业务。推动农业保险扩大范围、增加补贴。保障农民公平分享土地增值收益，鼓励社会资本投向农村建设，以镇村为单位加快完善基本公共服务体系，推进城乡要素平等交换和公共资源均衡配置。加快实施户籍制度改革，逐步把符合条件的农业人口转为城镇居民。推进区划调整，完成13个乡撤并改镇。抓好乡镇行政管理体制改革和小城镇综合改革试点工作。

　　坚持均等化取向，深化社会事业领域改革。扎实推进教育领域综合改革，优化学前教育管理体制，推进公办学校标准化建设，缩小城乡、校际差距，加大校长教师交流轮岗力度，扩大优质资源覆盖面，促进教育公平。做大做强特色文化产业，鼓励报业、广电、演艺集团市场化运作，引导社会资本多种形式投资文化产业，扶持开展对外文化交流，建立健全现代文化市场和公共服务体系。加快医药卫生体制改革，全面推进县级公立医院和基层卫生机构综合改革，巩固完善基本药物制度，推动医疗卫生信息资源共享、互联互通。建立完善统一的食品药品安全监管机构。推进机关事业单位养老保险制度改革，健全养老保险多缴多得激励和社会保险转移接续机制，出台城镇职工大病保险方案，健全社会保障财政投入和审计监督机制。

　　二、把握重大机遇，着力构筑新的开放优势。全面深入开展丝绸之路经济带建设年活动，强化载体建设，创新合作机制，在更大范围、更广领域、更高层次上推进对外开放。

　　提升港口服务功能。主体港区启动30万吨级航道二期工程，加快推进旗台作业区液体散货泊位建设。徐圩港区加快航道和防波堤工程建设，开工建设5万吨级通用泊位，建成通用散货和液体散货泊位一期工程。赣榆港区开工防波堤二期工程，建成10万吨级航道一期工程。灌河港区实施航道整治和燕尾作业区二期码头建设。加快北疏港道路和赣榆、徐圩港口铁路支线等集疏运体系建设。巩固提升一港两线运输效率。力争徐圩、赣榆口岸正式开放。

　　加快海陆双向开放。开工建设中哈过境货物运输中转分拨基地，争取设立中哈物流贸易园区，推进中韩陆海联运甩挂运输合作，强化新亚欧大陆桥铁路运输协调机制，进一步拓展面向日韩、连接中亚的物流经贸往来。办好通海国际物流博览会，加快建设国际展览交易中心和产业技术合作中心，争取设立大陆桥国际性会议永久会址。加强与中亚、日韩重要城市的友好交流，推动与阿拉木图等城市建立友好关系。推进互派办事处和商务机构，积极争取哈国铁路公司在连设立代办处。加强对中西亚、东北亚政治经济、民族文化的研究，支持高校加大语言教育和人才培养力度，鼓励互派留学生。主动融入上海自贸区建设，整合完善保税物流、出口加工、贸易服务等功能，争取设立综合保税区，积极推进自由贸易港区申报。

　　持续深化区域合作。强化与中西部地区的物流合作，促进陆桥沿线地区货源集聚，推进口岸管理部门跨区域信息互通、监管互认、执法互助。加快建设中西部地区出口产品生产基地和进口资源加工基地，建立完善产业合作利益分享机制，鼓励以区中区、园中园形式共建共用国家东中西区域合作示范区。探索建立丝绸之路经济带建设跨区域联动机制，促进政策沟通、道路联通、贸易畅通。深度融入长三角一体化，争取设立省加工贸易产业转移合作示范区，提升南北共建园区建设水平，努力在承接产业转移上实现更大作为。

　　强力推进招商引资。创新运作和激励机制，强化专业招商、产业链招商、以商引商，构建市县区联动招商格局。坚持境外重抓日韩港台新及欧美地区，境内重抓长三角、珠三角等制造业发达地区，办好连云港之夏、西游记文化节等大型经贸活动，强化招商活动成果的跟踪推进和项目督查。加强招商项目库建设，选准重点攻关项目，实行定点、定向、定人、定责招商，争取设立国家级重点台资产业合作示范区，力争对港台、日韩招商取得新进展，对世界500强企业、国内大型央企民企引资实现新突破。

　　积极稳定外贸增长。深入实施企业国际化战略，积极引导企业多元化开拓市场，健全外经贸联络协调制度，强化关贸、检贸、税贸、银贸合作，对重点外贸企业实行一企一策，新培育特色出口基地2个，创建省级以上出口品牌2个，完成外贸进出口总额69亿美元。鼓励有条件的企业开展境外投资合作，新增境外投资项目5个。推动侨务、对台、贸促工作再上新台阶。

　　三、注重质量效益，全面加快转型升级步伐。坚持稳中求进、以进促稳，利用经济稳定增长创造的空间，更大力度推进结构调整，打造连云港经济的升级版。

　　加快推动工业扩量提质。抓好100个新增产值5000万元以上新增长点项目，全社会工业投资增长21%，规模以上工业增加值增长13%，新增规模以上企业200家。加快发展临港产业。扎实推进炼化一体化项目各项规划编制、各类支持性文件完善、相关投资主体衔接、重大基础设施配套等工作，争取年内开工建设。加快富生石化、腾航铜业等重大项目建设，积极推进与华能、华电等央企项目合作，加快核电3、4号机组建设，新海电厂第二台1000兆瓦机组投产发电。化工、冶金产业产值分别突破1000亿元、750亿元。加快壮大新兴产业。加强规划引导和政策支持，组建产业发展联盟，推进新医药、新材料、新能源、装备制造等产业集群发展，三新产业产值超过1300亿元，装备制造业产值达到1200亿元。加大工业技改专项资金支持力度，着力扩大工业有效投入，实施重点产业技改项目100个，完成技改投资600亿元。加快中小企业园和工业集中区建设，完善邻里中心等配套服务设施，新建标准厂房300万平方米，招引培育特色项目500个。加大建筑业扶持力度，支持重点企业提升资质、壮大规模、打造品牌，建筑业增加值达到140亿元。

　　深入实施创新驱动战略。深化国家创新型试点城市建设，强化企业在技术创新中的主体地位，健全产学研协同创新机制。新建企业研发机构30个，培育高新技术企业30家，开发高新技术产品200个，实施重大创新与成果转化项目100项，高新技术产业产值突破1800亿元。增强科技创新平台公共服务能力，推进国家高性能纤维及复合材料高新技术产业化基地建设，市科技创业城一期全面建成投用。推动中科院能动中心申报建设国家重大科技基础设施，建成省海洋资源研究院海洋药物研发平台，建设中国药科大学连云港研究院、东华大学连云港纤维新材料研究院。落实质量强市战略。推进国家知识产权试点城市建设，专利授权量达2800件。推动连云港高新区升格。大力实施人才强市战略，引进高层次人才100人以上。

　　着力做大做强服务业。实施服务业创新百企示范工程，69个服务业重点项目完成投资137亿元，服务业增加值增长12%。依托金港湾国际物流园区，加快建设有色金属、煤炭、铁矿石等大宗商品交易市场，招引培育一批仓储物流企业，物流产业销售达700亿元。积极引进金融机构，加快推进江苏沿海开发银行总部和国家开发银行连云港分行组建工作，完成东海、灌南农商行组建。完成太平洋石英上市工作，支持企业到新三板挂牌融资。全年新增银行贷款180亿元、直接融资100亿元。大力发展休闲度假旅游业，加大配套设施建设力度，花果山创成国家5A级景区。加快发展文化产业，推进海峡两岸西游记文化创意中心、杰瑞科技创意产业园建设。提升商贸、房地产、中介等服务业，苏宁广场、嘉瑞宝国际广场建成开业。积极发展新兴服务业，推进物联网研究和应用，推动电子商务、云计算服务等新型业态成长。

　　加快发展现代农业。创新农业生产经营体制机制，灌云县整体完成承包土地确权登记颁证工作，全市新增土地流转20万亩。新建特色产业基地10个，新增市级农业龙头企业15家、家庭农场150个、农民专业合作社200家。各县区建设1至2个大型农产品批发市场，市级以上农业园区配套建设产地批发市场。推进农业标准化建设，制定推广农业基础设施、农产品品质提升等标准。建立耕地保护目标责任制，提升土地集约节约化利用水平，60%的耕地建成吨粮田，全面完成危仓老库改造，确保粮食安全。继续实施农田水利设施建设年行动计划，建设示范片区9个。基本实现水稻、玉米生产机械化，建成5万亩农业综合开发示范片2个、高标准农田20万亩。健全农产品检测网络，落实农产品产地编码和质量标识制度，逐步实现全程可追溯。

　　四、增强承载能力，加快推进新型城镇化进程。坚持以人为本，优化空间结构，加强功能建设，突出质态提升，统筹城镇化和新农村建设，着力构筑中心城市、县城和小城镇协调发展的城镇体系。

　　加快重点板块建设。新海新区加快推进新世界文化城三大街区、商务中心星海湖周边地块以及旅游精品商业街、游客服务中心建设，科创城公共服务中心、云计算中心、科技馆投入使用，全面建成两城两中心。连云新城加快西墅湾休闲街、新城学校、企业总部区等项目建设，让游客、居民、客商进得来、留得住。南部新城着力完善基础设施，加快科技创业产业园、现代服务业集聚区等重点载体建设。加快连云国际商务中心、新浦滨河新城等城市组团建设，推进快速公交、海滨大道等重点轴线两侧地块开发。加快完善商业办公、文化娱乐、卫生体育等城市公共配套，万达广场、昊海科技大厦等项目开工建设。完成旧城改造550万平方米，其中市区310万平方米，新建改造农贸市场8个，整治背街小巷260条，新增人防工程13万平方米，完成低洼片区改造10个。

　　强化基础设施配套。推进连盐铁路和204、310国道等重点交通工程建设，324、345省道建成通车。力争连徐客专列入国家中长期铁路网规划，争取连云港新机场、连淮扬镇铁路和连青铁路开工建设。完成大港路中期改造，打通科苑北路、运河路等6条断头路。加快推进花果山截洪沟、排淡河三期治理、临洪西泵站更新改造等项目建设，调整新海新区、连云新城河道水系，实施重点片区海堤达标工程，提升城市防洪排涝能力。完成蔷薇湖应急水源主体工程和饮用水输水工程。加大市政管网建设配套，新增供水、排污、燃气管网各30公里。新建9个110千伏以上输变电工程。加快城乡信息网络扩容提速，推进智慧港城建设。

　　打造山海相拥特色。建设云台山环山路和城市外环线，实现引山入城。海滨大道、徐圩港区港前大道基本建成，临海高等级公路全线通车，徐新公路主体贯通，实现揽山阅海。加强连岛、港口等重点地段的规划建设，推进环云台山周边、河流沿线、公园绿地综合整治。建成临洪水库，保护湿地生态，改善西盐河、龙尾河等城市河流水质，提升沿河绿化景观层次。保护修缮海州古城、南城古镇、板浦古镇等历史街区，延伸城市文脉。编制《市区城市色彩规划》，构建空间识别系统，彰显城市特色。

　　完善城市管理机制。深化四城同创，提高城市管理法治化水平，实现综合执法全覆盖。完善市、区、街道、社区四级城市管理体系，健全快速反应和长效管理机制，争创省优秀管理城市。加大城市环境综合整治力度，着力解决交通拥堵、抛洒滴漏、占道经营等突出问题。坚决查处违法用地和违章建筑，严格控制新的违建产生。建设数字房产，实现个人住房信息系统全市联网。切实加强物业规范管理，推进老小区整治工作，市区物业管理覆盖率达到86%。

　　重抓城乡统筹建设。建立健全城乡一体化规划管理体制，构建市区、县城、城镇、行政村、新社区五级规划体系，实现全市镇村规划全覆盖。四县县城进一步完善功能、彰显特色，提高集聚辐射和产业支撑能力。抓好临港、临海、临湖及沿交通干线的中心镇建设，培育壮大一批工业强镇、沿海重镇、现代农业大镇和乡村旅游名镇。完善试点镇村产业布局、基础设施配套，确保第一批试点全面完成、第二批基本达标。整治县乡河道231条。解决28万农村人口饮水安全问题。新建改建农村公路300公里，完成1800个村庄环境整治。

　　五、发展社会事业，坚持不懈增进民生幸福。推动一般就业向充分就业、劳有所得向劳有多得、学有所教向学有优教、病有所医向病有良医、住有所居向住有宜居、老有所养向老有颐养提升，推进文化惠民，实施新十大民生工程，让群众生活每年都有新改善，一年更比一年好。

　　稳步提高居民收入。深入推进省级创业型城市创建，完善创业培训、政策扶持、创业服务三位一体工作机制，实施创业助贷工程，成立创业助贷担保基金。多渠道增加就业岗位，完成各类职业技能培训25万人次，新增城镇就业8万人、农村劳动力转移就业3万人。保障农民集体经济组织成员权利，拓宽农民增加财产性收入渠道。健全工资决定和正常增长机制，推行企业工资集体协商制度，完善最低工资及支付保障制度。以石梁河库区扶贫为重点，深入推进新一轮脱贫工程，实现12万低收入人口、100个经济薄弱村达到省定脱贫目标。

　　加快发展社会事业。推进省教育现代化市级示范区建设，4个县区完成省学前教育改革发展示范区创建，2个县区通过国家义务教育均衡发展县区验收。改造中小学校舍30万平方米，加快建设海州高级中学、师专二附小新校区。强化省职业教育创新发展实验区和实训基地建设，创建省高水平现代化职业学校2所。推进高等院校内涵发展，加强合作办学，支持淮工积极发展研究生教育，推进财校升格创建和师专发展本科教育。开展健康港城促进行动，拓展区域卫生信息平台功能，推广使用居民健康卡，免费婚检率达75%以上。市公共卫生中心投入使用，市一院新海新区分院初步具备使用条件，市四院精神卫生中心主体封顶。实施文化惠民工程，完善四级文化服务网络，推进乡镇有线电视数字化整体转换，繁荣文化艺术创作，办好第八届中国曲艺节等节会活动，广播影视文化产业城投入使用。加快各区全民健身中心建设，建成十分钟体育健身圈，提高市体育中心使用效益和经营水平。加大计生奖励扶助力度，启动实施单独两孩政策。推动气象、科普、史志、档案、老区开发、关心下一代和红十字、老龄事业再上新台阶。

　　全面推进社保惠民。五险覆盖率稳定在95%以上。完善失地农民、失田盐民、失海渔民社会保障办法，将其统一纳入城乡社会保障体系。推行城乡居民大病保险，个人自付合规费用万元以上报销比例达到50%以上。新农合人均筹资标准提高到400元，住院费用报销比例提高到55%以上。新开工建设各类保障性住房5250套，改进住房公积金提取、使用、监管机制，发放廉租住房补贴840户。加强养老服务体系建设，新增养老床位2000张。落实低保标准增长机制，提高社会救助水平，发展慈善事业。建立健全单亲特困母亲、留守妇女、困境儿童、老年人、残疾人政策保障和关爱服务体系，切实维护弱势群体的权益。

加强社会治理创新。推进政府治理和社会自我调节、居民自治良性互动，培育发展行业协会、商会等社会组织，推进社会组织明确权责、依法自治、发挥作用。完成村委会、居委会换届选举。健全基层综合服务管理平台。扎实开展法治连云港、平安连云港建设。完善重大决策社会稳定风险评估、信访事项依法终结和群众满意度评价等机制，有效预防化解社会矛盾。加强社会治安综合治理，严防各类违法犯罪活动。加强重点领域安全监管，着力构建安全生产目标责任、执法监督、科技支撑、基层基础四大体系。严格食品药品全过程安全监管，建成投用市食品药品检验检测所。强化应急管理和防灾减灾工作，做好校园、校车安全保障工作。依法管理民族宗教事务。加强国防动员、双拥共建和民兵预备役工作，争创全国双拥模范城七连冠。加强精神文明建设，努力营造安全稳定、文明和谐的社会氛围。

　　六、保护资源环境，大力推进生态文明建设。推进绿色发展、循环发展、低碳发展，努力把环境敏感期变成生态建设加速期，让港城的山更绿、水更清、天更蓝。

　　切实加强环保基础工作。严格落实环境准入制度，完善项目环评、规划环评审批联动和责任追究机制。加快推进化工园区污水处理厂、配套管网和集中供热设施建设，完成临港化工园区卫生防护距离内居民搬迁。完善城乡生活垃圾统筹处理体系，建成刘湾垃圾填埋场。落实最严格的水资源管理制度，强化集中式饮用水源地达标建设，着力提高城镇污水收集和处理效率，建成15个乡镇污水处理厂。

　　全面开展环境综合整治。深入整治蔷薇河沿线涉氟及酸洗企业，开展农业面源污染治理。坚持堵疏结合、以禁促用，建立秸秆综合利用和禁烧工作长效机制，全面推进多种污染物协同减排，努力解决大气污染突出问题。加强风景名胜区、废弃矿区水土流失综合治理，严厉打击非法开山采石，完成市区7个废弃塘口整治复绿。推进工业企业治污工程，改造提升化工园区，完成节能改造项目35个、清洁生产项目15个。

　　大力提升生态建设水平。深入实施生态市建设规划，完成30个省级以上生态乡镇创建，力争东海县、赣榆县建成国家级生态县。调整优化重要生态功能区，完成市县级海洋功能区划修编，推进近岸海域、海岛生态修复和海州湾国家级海洋公园建设。广泛开展低碳全民行动，深入推进公共机构节能工作。以创建全国绿化模范城市为契机，大力开展云台山等重点片区绿化，新增城市绿地400公顷、沿海防护林4万亩，完成绿化造林15万亩，全市林木覆盖率达到28.1%。

　　七、加强政府自身建设，树立为民务实清廉形象。深入开展党的群众路线教育实践活动，切实加强政府治理，进一步转作风、提效能、塑形象，努力建设人民满意的法治政府和服务型政府。

　　深化审批制度改革。大力开展减事项、减环节、减收费三减行动，实施前置审批联用、相关事项并联审批、园区前置审批联办、项目竣工联合验收四联审批。对审批事项和环节能减尽减、行政权力能放则放，确保审批事项减少到100项以内，基本建设审批用时控制在100天以内，审批事项集中率达100%，创造更加宽松的发展环境。

　　严格规范行政行为。牢固树立法治观念，优化职能配置，规范权力运行，严格按照法定权限和程序行使权力、履行职责。完善重大问题集体决策、专家咨询制度，健全重大事项向人大报告制度，对重大决策实行合法性审查、风险评估、跟踪问效。规范行政执法行为，加强执法监督检查，抓好重点领域专项治理。全面推进政务公开，有序开展财政预决算和审计结果公开，主动接受社会和舆论监督。

　　改进机关工作作风。严格落实岗位责任制、服务承诺制、首问负责制、限时办结制，切实提高机关服务效能。优化绩效管理，突出体现转型升级、生态建设、民生改善目标导向。强化重要决策部署和重大事项推进的督查落实，建立督查结果与目标考核挂钩机制，严格行政问责，确保政令畅通，切实提高政府执行力和公信力。

　　树立清正廉洁形象。全面落实中央八项规定和省、市委的各项规定，坚决做到令行禁止。严格执行审计、监察、财政、国有资产监管等制度，完善公共资源交易管理体系，确保让权力在阳光下运行。继续贯彻落实国务院约法三章规定，大力倡导勤俭节约，坚决反对铺张浪费，严控三公经费支出。深化廉政文化建设，筑牢思想道德防线，保持清正廉洁、为民务实的人民公仆本色。

　　各位代表，形势催人奋进，使命艰巨光荣。时不我待，只争朝夕。让我们在市委的坚强领导下，团结和带领全市人民，统一思想、凝聚共识，锐意进取、扎实工作，为建设幸福港城、美好家园而努力奋斗！
